# Calcifying vascular smooth muscle cells and osteoblasts: independent cell types exhibiting extracellular matrix and biomineralization-related mimicries

**DOI:** 10.1186/1471-2164-15-965

**Published:** 2014-11-07

**Authors:** Rodrigo DAM Alves, Marco Eijken, Jeroen van de Peppel, Johannes PTM van Leeuwen

**Affiliations:** Department of Internal Medicine, Erasmus MC, Wytemaweg 80, 3015 CN Rotterdam, The Netherlands

**Keywords:** Calcifying, Vascular smooth muscle cells, Osteoblasts, Gene expression, Extracellular matrix, Biomineralization

## Abstract

**Background:**

Ectopic vascular calcifications represent a major clinical problem associated with cardiovascular disease and mortality. However, the mechanisms underlying pathological vascular calcifications are largely unknown hampering the development of therapies to tackle this life threatening medical condition.

**Results:**

In order to gain insight into the genes and mechanisms driving this pathological calcification process we analyzed the transcriptional profile of calcifying vascular smooth muscle cells (C-VSMCs). These profiles were compared to differentiating osteoblasts, cells that constitute their physiological calcification counterparts in the body. Overall the transcriptional program of C-VSMC and osteoblasts did not overlap. Several genes, some of them relevant for bone formation, were distinctly modulated by C-VSMCs which did not necessarily lose their smooth muscle cell markers while calcifying. Bioinformatics gene clustering and correlation analysis disclosed limited bone-related mechanisms being shared by two cell types. Extracellular matrix (ECM) and biomineralization genes represented common denominators between pathological vascular and physiological bone calcifications. These genes constitute the strongest link between these cells and represent potential drivers for their shared end-point phenotype.

**Conclusions:**

The analyses support the hypothesis that VSMC trans-differentiate into C-VSMCs keeping their own identity while using mechanisms that osteoblasts use to mineralize. The data provide novel insights into groups of genes and biological processes shared in MSC and VSMC osteogenic differentiation. The distinct gene regulation between C-VSMC and osteoblasts might hold clues to find cell-specific pathway modulations, opening the possibility to tackle undesired vascular calcifications without disturbing physiologic bone formation and *vice versa*.

**Electronic supplementary material:**

The online version of this article (doi:10.1186/1471-2164-15-965) contains supplementary material, which is available to authorized users.

## Background

Vascular calcification in the tunica media of arteries and blood vessels is often observed in the elderly population, in patients with diabetes mellitus and/or chronic kidney disease [1]. Vascular calcifications represent a major clinical problem being in the origin of cardiovascular disease and ultimately mortality [2]. Vascular smooth muscle cells (VSMCs) are contractile cells located at the medial layer of the vessel wall. VSMCs can be triggered to transdifferentiate into calcified vascular cells (C-VSMCs), loosing the phenotypic markers responsible for smooth muscle cell contractility [3, 4]. Further physiological alterations of VSMC include entering a synthetic state with abundant production of extracellular matrix (ECM) proteins [1] followed by matrix vesicle-mediated calcification [5, 6].

It has been hypothesized that pathological medial calcification is a process analogue to bone mineralization with VSMCs entering an osteoblast-like differentiation program [7]. Atherosclerotic plaques, of medial and valvular origin, express several bone-related ECM proteins, including osteopontin, collagen I, matrix GLA protein (MGP), osteonectin and osteocalcin [7–9]. In addition, calcified vascular tissue expresses bone specific transcription factors and bone morphogenetic proteins (BMPs) [10–13]. Despite these similarities with osteoblast differentiation the exact mechanism behind VSMCs transdifferentiation into C-VSMCs remains largely unknown. Some studies have suggested that only a subset of the VSMC pool has osteogenic potential [10, 14]. Pathological vascular calcifications may arise due to loss of mineralization inhibitors, which are continuously expressed in healthy vascular tissue [15]. Mice lacking MGP show spontaneous vascular calcifications [8], a phenotype that is exacerbated when SPP1, another mineralization inhibitor, is deleted [16]. Murshed and colleagues [17] have explored this hypothesis further showing that mineralization can occur in any collagen type I rich tissue that expresses pyrophosphatases such as alkaline phosphatase (ALP). While collagen type I is ubiquitously expressed in the tissues, the co-expression of this ECM protein with ALP is restricted to those that mineralize. ALP is involved in the cleavage of pyrophosphate a potent mineralization inhibitor [18]. This enzyme on its own was shown to be able of inducing calcification in rat models of medial calcification [19]. Normally VSMCs do not express ALP but for unclear reasons they can transdifferentiate into C-VSMCs that show increased ALP activity [6, 20].

In this study we aimed to reveal the processes whereby VSMCs develop into C-VSMCs exhibiting a calcified phenotype. We compared this pathological process to the physiological mechanism regarded as an analogue process, the differentiation of mesenchymal stem cells into osteoblasts.

Under consideration were three hypotheses, 1) C-VSMCs are osteoblast or osteoblast-like cells transdifferentiating from the VSMC pool, 2) C-VSMCs initiate mineralization using osteoblast-like mechanisms, and 3) C-VSMCs mineralize using mechanisms unrelated to osteoblasts. To address these hypotheses we used genome-wide gene expression analysis during *in vitro* human VSMC development into C-VSMCs and human mesenchymal stem cell (MSC) differentiation into osteoblasts. We investigated these processes in terms of their known specific markers but also in an unbiased general perspective, using bioinformatics tools. Global expression profiles and gene regulation were used to pinpoint the transcriptional program and the identity of a C-VSMC in comparison to the phenotype-resembling osteoblast.

## Results

### The complete VSMC population develops into an ALP positive population under osteogenic stimuli

**Figure 1:**
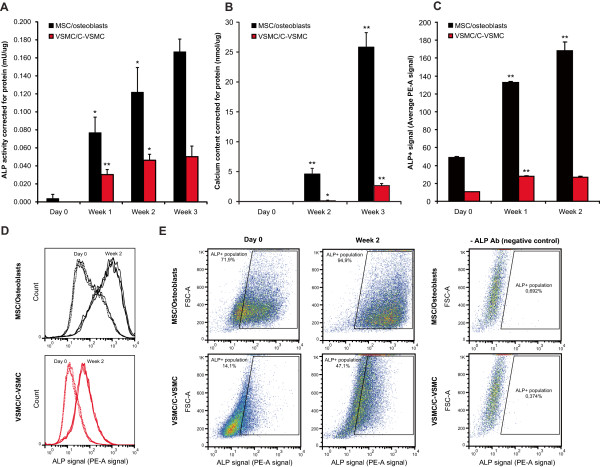
**Characterization of the C-VSMC development and osteoblast differentiation processes.** ALP activity **(A)** and mineralization **(B)** corrected for protein during the 3 week cell culture period. ALP + cell signal, measured by FACS until the second week of culture, is shown in panel **(C)**. Detailed scatter plots with the distribution of the ALP + signal between the cell populations are depicted in **(D)** and **(E)**. Value means ± SD (n = 3, *p < 0.05, **p < 1×10^-4^).

VSMCs and MSCs were cultured in osteogenic medium for 25 days to induce development into C-VSMCs and osteoblast respectively. During this period total ALP activity was measured. As shown in Figure 1A, ALP activity increased in C-VSMCs and osteoblasts cultures compared to their precursor cells with enzymatic activity reaching higher absolute levels in osteoblasts than in their C-VSMC counterparts.

In addition, we measured ALP expression at the individual cell level by flow cytometry. This data (Figure 1C) corroborated the ALP activity measurements. Furthermore it demonstrates that MSC and VSMC (trans) differentiation is characterized by an expansion of the ALP + cell pool (Figure 1D and E).

### C-VSMCs and osteoblasts have distinct global gene expression profiles

**Figure 2:**
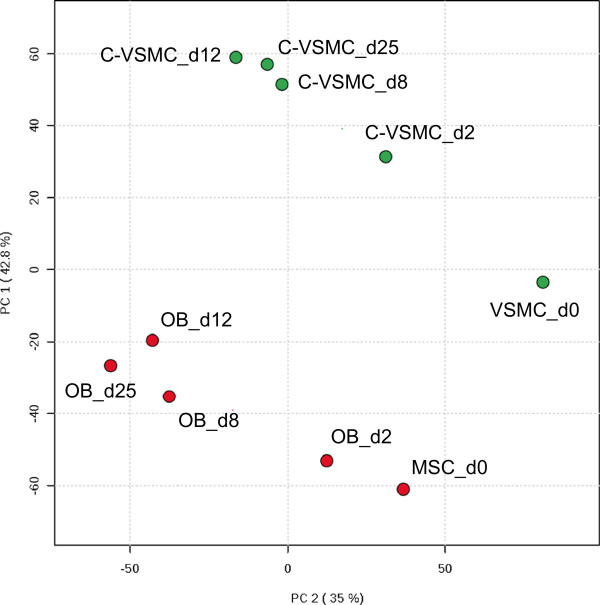
**Principal Component Analysis of the global gene expression changes occurring during C-VSMC development and osteoblast differentiation.** 14733 probes expressed by both VSMC/C-VSMC and MSC/osteoblasts (OB) at day 0, 2, 8, 12 and 25 were considered for analysis. Distance between samples is directly proportional to gene expression differences. Each time point is represented by the average of 3 biological replicates with exception for day 0 where n = 4. Between parenthesis in the x- and y-axis is the percentage of variance captured by the two principal components.

Next, we performed comparative genome-wide mRNA expression analysis in osteogenic VSMC and MSC cultures to characterize their transcriptional similarities and dissimilarities. Five time-points (day 0, 2, 8, 12 and 25) were analyzed during VSMC development to C-VSMCs and MSC to osteoblasts. The data were normalized and probes/genes expressed in neither VSMC/C-VSMC nor MSC/osteoblasts were excluded from further analysis. The overlap of expressed probes between osteogenic VSMC and MSC cultures contained 14733 probes representing 11302 unique genes. These probes/genes were subsequently used for Principle Component Analysis (PCA). PCA allowed simultaneous comparison of multiple time-points in both cell types summarizing the relationship between them. The closer the data points appear in the PCA plot (Figure 2), the more similar their gene expression profiles are. The PCA plot showed that VSMCs and MSCs at the start of culture (day 0) represented two clearly distinct clusters that upon osteogenic stimulation did not converge into an indistinguishable cluster of similarity (Figure 2). In other words, C-VSMCs and osteoblasts are two distinct cell types in terms of global gene expression.

Several clusters could be identified during C-VSMC and osteoblast development. For both cell types, day 2 represented an intermediate stage after the osteogenic stimuli given to VSMCs and MSCs (day 0; Figure 2). This transient stage is followed by a more stable period, day 8-25, in which gene expression did not change so dramatically (Figure 2).

### VSMC calcifications are not dependent on the down-regulation of smooth muscle cell contractile markers

**Figure 3:**
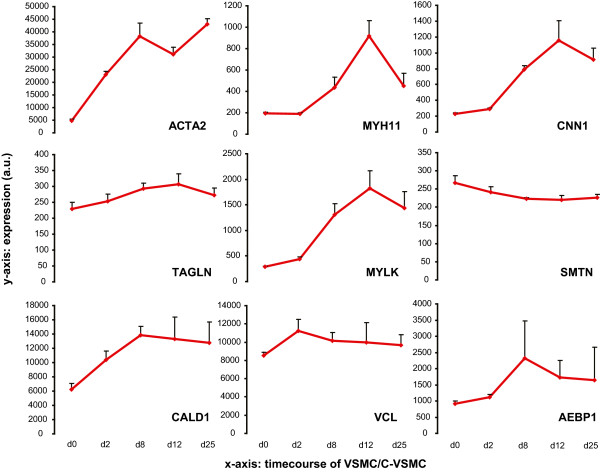
**Expression profile of known smooth muscle cell markers during C-VSMC development.** Intensity values in arbitrary units are based on data at day 0, 2, 8, 12 and 25. For each time point n = 3 with exception for day 0 where n = 4. Value means ± SD.

In the subsequent analysis we investigated the expression of (vascular) smooth muscle cell marker genes. We selected established VSMC markers described in literature [21], including alpha-actin-2 (ACTA2), smooth-muscle myosin (MYH11), calponin (CNN1), smooth muscle protein 22-alpha (TAGLN), telokin (MYLK), smoothelin (SMTN), caldesmon (CALD1), vinculin (VCL) and adipocyte enhancer-binding protein 1 (AEBP1) (Figure 3). We verified that expression of many of these genes was increased in C-VSMCs compared to their VSMC precursors during osteogenic conditions. This result was confirmed by qPCR but it could not be replicated in C-VSMCs from a second independent donor (Additional file 1: Figure S3). This data demonstrate that C-VSMC are able to transdifferentiate without losing the contractile phenotype markers of VSMC. In addition it raises the idea C-VSMC do not necessarily acquire a full osteoblast-like transcriptome, something also found to be true for other models of vascular calcification [22].

### Genes identically regulated by C-VSMCs and osteoblasts are functionally annotated to extracellular region

To identify whether only specific groups of genes were identically regulated by C-VSMCs and osteoblasts, we have selected differentially expressed genes during VSMC development into C-VSMCs and during MSC differentiation into osteoblasts. Differential expression was calculated for each cell type relative to day 0, when osteogenic treatment was initiated. Probes/genes were considered differentially expressed when on at least one day during culture their log_2_ fold-change compared to day 0 was significantly (q-value < 0.001) higher than 0.5 (up-regulation) or lower than -0.5 (down-regulation). During C-VSMC development and osteoblast differentiation 3721 probes and 3114 probes met this criterion, respectively. Considering the two cell types combined, 4782 probes were found to be differentially expressed (Additional file 2: Table S1). Of these 4782 probes, 1638 and 1061 were exclusively differentially expressed in C-VSMCs or in osteoblasts, respectively. Regarding the direction of gene expression regulation, 1968 probes were identically regulated while 150 were oppositely changed during C-VSMC and osteoblast development (Additional file 2: Table S1).

**Figure 4:**
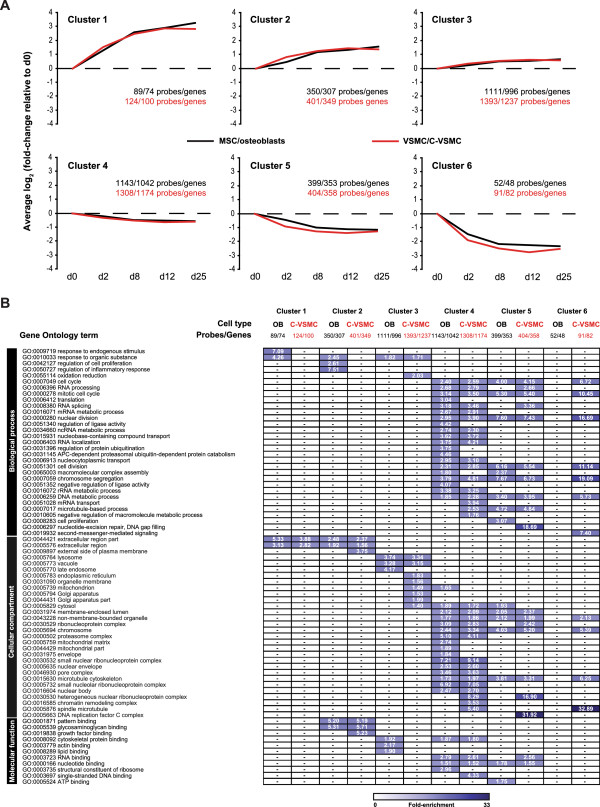
**Clustering of genes with similar expression patterns in C-VSMCs and osteoblasts and respective functional annotation of the clusters. (A)** Clusters of genes with similar expression pattern for C-VSMCs and osteoblasts were obtained using k-means clustering (k = 6). For these analysis only differentially expressed genes were used. Average relative gene expression level (log_2_ fold-change relative to day 0) for all probes within each cluster at the different time points analyzed is shown. **(B)** Functional annotation for each of the 6 clusters in C-VSMCs and osteoblasts. Only significant (Bonferroni p-value < 0.05) biological process, cellular compartment and molecular function annotations were considered for analysis. Numbers within grid represent fold-enrichment levels of GO-terms in the distinct clusters. The number of probes/genes comprised in each cluster is also indicated.

The temporal and directional expression dynamics of the 4782 differential expressed probes during C-VSMC development and osteoblast differentiation is resumed in Figure 4. K-means clustering separated the differentially expressed probes during C-VSMC development and osteoblast differentiation into clusters sharing common regulation patterns. On basis of Figure of Merit (FOM) analysis we concluded to divide gene expression data in 6 clusters (Figure 4A). This number of clusters was found to provide good predictive power for the k-means algorithm (Additional file 3: Figure S1) without restricting the cluster size for functional annotation analysis. Functional Gene Ontology (GO) annotation of genes underlying these clusters revealed information about the biological processes, cellular compartments and molecular functions during C-VSMC development and osteoblast differentiation (Figure 4B).

Clusters 1, 2 and 3 contained up-regulated genes while clusters 4, 5 and 6 represented down-regulated genes in both C-VSMCs and osteoblasts (Figure 4A). In clusters 1 and 2 C-VSMCs and osteoblast shared the over-representation of genes linked to extracellular region (GO:0044421 and GO:0005576, Figure 4B). In clusters 3, 4, and 5 several GO-terms were also shared by C-VSMCs and osteoblasts but these were more general GO-terms like cell cycle, RNA processing, chromosome, biological response to organic substance, etc., related to general cell function/metabolism. An exemption was cluster 6 that only showed significant enriched GO terms for C-VSMCs. This fact may be attributed to statistical issues related to the lower number of genes fitting this cluster in osteoblasts. Overall, cluster analyses did not clearly identify sets of bone-related processes or cellular components shared by C-VSMCs and osteoblast. Nevertheless, it was interesting to observe that a common set of extracellular region genes from cluster 1 and 2 (Figure 4A and B) was similarly regulated by both cell types indicating a shared mechanism involving changes in the extracellular environment/matrix.

### C-VSMCs express a subset of extracellular matrix genes and genes involved in biomineralization

**Figure 5:**
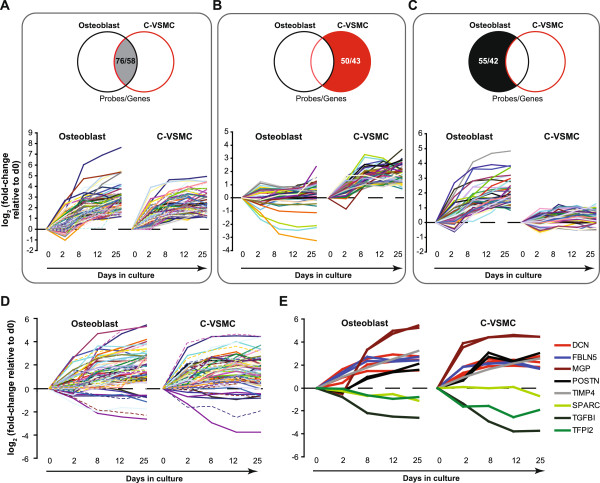
**Expression pattern of extracellular region and ECM genes differentially expressed during C-VSMC development and osteoblast differentiation.** Temporal expression profile of extracellular region and ECM genes shown in Figure 4 clusters 1 and 2 in **(A)** both cell types, **(B)** in C-VSMCs only and **(C)** in osteoblasts only. Numbers in the Venn diagrams indicate number of probes/genes. **(D)** Expression profile of ECM probes/genes with identical regulation pattern in C-VSMCs and osteoblasts. A smaller subset of these ECM genes is shown in **(E)**. Expression is plotted as log_2_ fold-change relative to d0. Each line plotted represents a probe set. Probe/gene identifiers are provided in Additional file 4: Table S2 and Additional file 5: Table S3.

Considering the relevance of the extracellular environment for osteoblast differentiation and mineralization, we analysed in greater detail the expression of genes linked to extracellular region present in cluster 1 and 2. Cluster 1 and 2 contained in total 58 extracellular region genes (equivalent to 76 probes; Additional file 4: Table S2) overlapping in C-VSMC and osteoblasts (Figure 5A). Expression pattern analyses of the cell type-specific genes from cluster 1 and 2 (43 and 42 for C-VSMCs and osteoblasts respectively; Additional file 4: Table S2) showed clearly distinct expression patterns for C-VSMCs (Figure 5B) and osteoblasts (Figure 5C).

The observation that among the 58 extracellular region genes overlapping in C-VSMCs and osteoblasts was a large subpopulation of ECM genes prompt us to identify differentially expressed ECM genes (GO:0031012) identically modulated in both cell types. From the 126 (160 probes) differentially expressed ECM genes in total 57 (76 probes) were identically modulated in C-VSMCs and osteoblasts (Additional file 5: Table S3). The expression pattern of these 57 genes is shown in Figure 5D. Some of them are known to be involved in mineralization process of both the bone and the vasculature. This is the case for POSTN (periostin) and ADAMTS1 (ADAM metallopeptidase with thrombospondin type 1 motif), two genes showing consistent regulation across distinct primary cell donors (Additional file 1: Figure S3).

**Figure 6:**
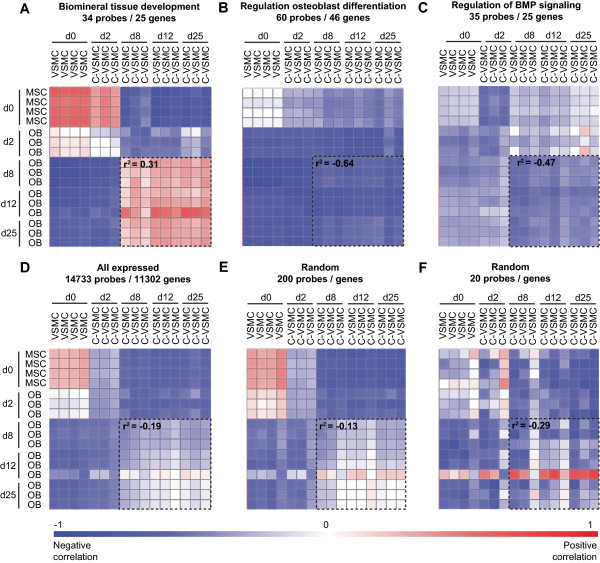
**Pearson correlation plot of genes comprised within Gene Ontology (GO)-terms related to bone biology.** VSMC/C-VSMC and MSC/osteoblast are plotted against each other to determine their degree of similarity based on **(A)** biomineral tissue development, **(B)** regulation of osteoblast differentiation and **(C)** regulation of BMP signaling genes. As a reference, VSMC/C-VSMC and MSC/osteoblast are also plotted considering **(D)** all expressed genes and **(E-F)** randomly selected genes. Dashed boxes highlight the correlation between C-VSMCs and osteoblasts at day 8-25. Average correlation values (r^2^) for this group of samples is shown within the dashed boxes; blue, negative correlation; red, positive correlation.

In an alternative approach to compare osteoblasts and C-VSMC we performed gene correlation analyses based on a priori selected GO-terms that are relevant for bone formation and mineralization. These GO-terms included among others biomineral tissue development, osteoblast differentiation and regulation of BMP signaling genes (see the full list of GO-terms analyzed in the Material and Methods section 2.7). To assess specificity, correlation analyses were also performed for a randomly selected set of expressed genes. Genes involved in biomineral tissue development (25 genes; Additional file 6: Table S4) showed the highest correlation between C-VSMCs and osteoblasts (r^2^ = 0.31; Figure 6A) with an r^2^ much higher than for a similar number of randomly selected expressed genes (r^2^ = -0.29; Figure 6E). On the contrary, GO term such as regulation of osteoblast differentiation and BMP signaling failed to correlate C-VSMCs and osteoblasts (Figure 6B and C). In Additional file 7: Figure S2 the expression pattern of a selection of genes driving the correlation in the GO-term biomineral tissue development and the anti-correlation in the GO-term regulation of BMP signaling is shown. At least LEP (leptin) and SOST (sclerostin), from the correlation and anti-correlation group of genes respectively, could be validated distinct donors (Additional file 1: Figure S3). Moreover, we found good translation between leptin transcript and protein levels, especially for the donor used in the array (Additional file 3: Figure S3). Altogether, our results demonstrate that a specific subset of extracellular genes, including ECM genes, together with genes involved in the regulation of mineralization represent a common denominator between C-VSMCs and osteoblasts.

## Discussion

The current comparative global gene expression profiling analyses of osteoblasts and C-VSMC demonstrate that VSMC under an osteogenic stimulus only partially mimic osteoblasts. Despite the fact that C-VSMCs had an overall transcription profile distinct from osteoblasts, the two cell types regulated identically subsets of ECM and biomineralization genes. These results support the hypothesis that VSMCs require specific osteoblast-related gene modulation and mechanisms to transdifferentiate into C-VSMCs.

The mechanisms responsible for the transformation of a contractile VSMC into a stiff, mineral surrounded cell are still poorly understood. We demonstrated that the whole VSMCs pool has osteogenic potential and progresses towards ALP + cells when exposed to osteogenic stimuli. This indicates that C-VSMCs are not derived from a small and specific vascular cell subpopulation with osteogenic potential, as shown in other vascular calcification models [10, 14]. The relatively homogenous C-VSMC population (based on ALP activity) observed in our study enabled us to use global gene expression profiles.

Genome-wide gene expression analyses in *in vitro* models of pathological and physiological mineralization revealed important characteristics of vascular calcifications. C-VSMC development (and osteoblast differentiation) comprised three major phases. The first phase contained VSMC before being triggered to transdifferentiate (day 0; Figure 2). When VSMC were exposed to an osteogenic stimulus, their transcriptional program was quickly altered entering a transient intermediary stage (day 2; Figure 2) after which transcriptional changes became more subtle (day 8-25; Figure 2). We believe that the intermediary phase represents a commitment period responsible for the transition of VSMC into C-VSMCs. In this respect, the clusters of genes identified to be down-regulated (cluster 4-6; Figure 5) were particularly interesting due to their annotation to GO-terms involved in the regulation of cell cycle, cell division and transcription. We believe that the modulation of genes with such functions is possibly associated to the switch of VSMC from proliferative into transdifferentiating cells [3, 4], an effect observed in osteoblasts under the influence of glucocorticoids [23, 24].

Regarding the comparative transcription profiling, we identified 57 ECM genes identically regulated by C-VSMCs and osteoblasts. Their common modulation pattern strongly supports their structural and/or regulatory role in both forms of calcification. On the other hand, ECM genes that did not share identical expression in both cell types are likely to be less crucial for mineral deposition. However, ECM gene data are yet difficult to interpret since little is known about the function of most of the proteins encoded by these genes in matrix mineralization. Genes like DCN, MGP and POSTN constitute exceptions, being known for their crucial role during bone formation and mineralization [8, 25, 26]. MGP for example is a potent inhibitor of calcification both in bone and in the vasculature [8]. These genes represent a strong evidence for the implication of the other ECM genes, with yet unknown relationship to matrix mineralization, in vascular calcifications. This evidence is further substantiated by the fact that several ECM genes (e.g. FBLN5, POSTN, TIMP4) are targets of activin A, a potent inhibitor of ECM mineralization [27]. Recently we have identified over 1200 different proteins present in bone tissue [28]. It is conceivable that ECM proteins act in concert with each other and that the combination of ECM proteins eventually determines the extent of mineralization. It will be a great challenge to identify and characterize these interactions. The current study demonstrating only a limited overlap in ECM gene expression between osteoblasts and C-VSMC will facilitate this challenge by enabling to focus on the selection of overlapping ECM genes. Additional studies focusing on this subset of genes are essential to prove their involvement in biomineralization and during the atherosclerotic process in particular.

Besides ECM, analysis of genes differentially regulated during physiological and pathological calcifications revealed that C-VSMCs share specific genes related to the GO-term biomineral tissue development. ALPL (alkaline phosphatase), GPNMB (glycoprotein nmb), LEP (leptin), PTN (pleiotrophin) and SRGN (serglycin) were among genes within this GO-term that have been already studied in the context of tissue calcifications. ALPL has been shown to be fundamental for mineralization. This pyrophosphatase inactivates the mineralization inhibitory pyrophosphate [29] facilitating not only bone but also vascular calcifications [17, 19]. Together with ALPL, LEP and GPNMB are genes capable to promote calcifications. LEP is an energy metabolism hormone that enhances mineralization both in bone [30, 31] and in vascular tissue [32] while GPNMB, is a glycoprotein implicated in end-stage renal disease (ESRD) a pathological condition associated to ectopic calcifications [33]. Biomineral tissue development genes did not include only genes favouring mineral deposition. SRGN was recently described as an inhibitor of osteoblast mineralization [34]. Despite not described with respect to ectopic mineralizations, the up-regulation of this gene during C-VSMC development might represent a mechanism to protect the vasculature from calcifications similarly to what is described for MGP [15].

Our comparative gene expression profiling constitutes a powerful tool to identify novel targets to control physiological as well as pathological calcifications. Nevertheless, our bioinformatics approach was limited to the identification of genes currently annotated in GO databases as belonging to ECM or involved in biomineralization. We hypothesize that more ECM and biomineralization genes are involved in both forms of calcification but because they are not yet annotated as such they were missed in our analysis. A limitation of our approach is related to the heterogenic response across primary cell sources [35] leading to distinct temporal dynamics during differentiation and mineralization. Overall, results obtained by gene expression array could be confirmed using qPCR within the same donor (Additional file 1: Figure S3). However, analysis of 2 distinct donors revealed less consistent results, likely due to the natural variability of these primary cells and their donors [36].

Correlation analysis of bone-related genes expressed during VSMC transdifferentiation showed groups of genes negatively correlated between C-VSMCs and osteoblasts, substantiating the uniqueness of the former cell type. For example, genes of the important osteoblast BMP/TGF-β/Activin signaling cascade (e.g. ACVR2A, GREM1, SMAD7) were oppositely regulated by C-VSMCs and osteoblasts. The divergence of these genes in C-VSMCs and osteoblasts supports the concept of cell-specific pathway modulations in both cell types. This is something recently observed in other tissues/cells [37, 38] but not yet investigated with respect to medial vascular calcifications and bone. Nevertheless, BMP7 is a gene that appears to corroborate this concept since it is described as promoter of normal osteoblast function [39, 40] and capable to prevent atherosclerosis [41, 42]. More studies are needed to define the exact role of each of these genes and most importantly their cross-talk to other signaling pathways [43, 44], like the Wnt signaling of which we have identified genes distinctly modulated between C-VSMCs and osteoblasts (e.g. SOST).

## Conclusions

Altogether, the different analyses support the hypothesis that VSMC transdifferentiate into C-VSMCs keeping their own identity while using mechanisms that osteoblasts use to mineralize. Extracellular (matrix) genes and genes involved in tissue mineralization constitute important common denominators between pathological vascular and physiological bone calcifications. A limitation of our study is that one still has to study heterogeneous MSC and VSMC populations that differ from donor to donor in magnitude of gene expression and temporal dynamics, which relates to differences between donors we observed. Nevertheless the current study provides novel insights into groups of genes and biological processes shared in MSC and VSMC osteogenic differentiation. Our data ought to be tested in a wider pool of primary cell donors in order to further discriminate the consistently regulated genes. Finally, distinct gene regulation between C-VSMC and osteoblasts might be of interest to find cell-specific pathway modulations, opening the possibility to tackle undesired vascular calcifications without disturbing physiologic bone formation and *vice versa*.

## Methods

### Cell culture

Human bone marrow-derived Mesenchymal Stem Cells (MSCs; PT-2501, Lonza, Walkersville, MD, USA) and Vascular Smooth Muscle Cells (VSMCs; coronary artery smooth muscle cells, CC-2583, Lonza) were cultured as described previously [27]. Briefly, MSCs and VSMCs were expanded in Mesenchymal Stem Cell Basal Medium (MSCBM, PT-3238, Lonza) supplemented with Mesenchymal Stem Cell Medium SingleQuot Kit (MSCGM, PT-4105, Lonza) and Smooth muscle cell Basal Medium (SmBM, CC-3181, Lonza) supplemented with Smooth muscle Medium-2, SingleQuot Kit (SmGM-2, CC-4149, Lonza) respectively. For induction of MSCs differentiation into osteoblasts (referred also as MSC/osteoblasts) and VSMC development into C-VSMCs (VSMC/C-VSMC), cells were cultured in DMEM medium (GIBCO, Paisley, UK) containing 10% FCS, penicillin/streptomycin, 1.8 mM CaCl_2_ (Sigma, St. Louis, MO, USA) and 20 mM HEPES (Sigma), pH 7.5. Additionally, this medium was freshly supplemented with 0.1 mM ascorbic acid (Sigma), 10 mM ß-glycerophosphate (Sigma) and 100 nM dexamethasone (DEX, Sigma). In the present study 2 independent MSC and VSMC donors were used, one for the gene expression array and the other for validation purposes. All analyses were performed on samples collected at the beginning of cell culture (day 0, before induction of differentiation) and during week 1, 2 and 3 of culture.

### Alkaline Phosphatase and protein concentration

ALP activity was assayed as described elsewhere [45]. Results were corrected for the protein content of the cell lysates. Protein concentration was determined using a BCA kit (Pierce Biotechnology, Rockford, IL, USA) following the manufacturer’s instructions.

### Flow cytometry analysis of ALP positive cell population

Cells were washed in PBS, trypsinized and fixed in 2% PFA for 10 min at room temperature. Cells were permeabilized in 90% ice-cold methanol and after re-suspension incubated for 10 min in blocking solution (PBS/0.5% BSA). Cells were probed with a primary monoclonal mouse antibody against Alkaline Phosphatase, Tissue Non-Specific (1:100, 1 h; ab17973, Abcam). A goat anti-mouse IgG R-Phycoerythrin conjugated antibody (1:50, 30 min; M30004-1, Invitrogen, Camarillo, CA, USA) was used as a secondary antibody. Finally, cells were re-suspended in PBS and the ALP + population was measured in the PE-A channel (excitation 488 nm) using a Becton Dickinson FACS-Canto (BD Biosciences).

### RNA isolation and quantification

Total RNA was isolated using TRIzol (Invitrogen) according to the manufacturer’s instructions. An additional step was introduced to remove calcium (derived from ECM). RNA was precipitated by overnight incubation with 4 M LiCl and 50 mM EDTA at −20°C. After precipitation and centrifugation for 30 min at 14,000 rpm and 4°C, the RNA pellet was washed four times with 70% ethanol and dissolved in H_2_O. The RNA concentration was determined spectrophotometrically using a NanoDrop ND-2000 (Thermo Scientific, Wilmington, DE, USA) and its quality accessed by RNA 6000 Nano assay on a 21000 Bioanalyzer (Agilent Technologies, Santa Clara, CA, USA), both according to the manufacturer’s instructions.

### Illumina gene chip-based expression

Gene-chip based expression was performed essentially as recently described [46] using 3 biological replicates per condition with exception for day 0 cultures for which 4 replicates were used. Briefly, 150 ng of RNA were amplified using the Illumina TotalPrep RNA Amplification kit (Ambion, Austin, TX, USA) as recommended by the manufacturer. Single-stranded cDNA was generated using a T7 oligo(dT) primer and was followed by second-strand cDNA synthesis. cDNA was further transcribed *in vitro* using a T7 RNA polymerase generating biotin-labeled cRNA. After cRNA purification its quality was checked on a Bioanalyzer (Agilent Technologies) and its concentration determined using a NanoDrop (Thermo Scientific). Per array, 750 ng of cRNA were hybridized, washed and detected using the standard Illumina protocol. Slides were scanned on an iScan and analyzed using Genome Studio v2010.1, both from Illumina.

### Gene expression data processing

Gene expression data were processed as described elsewhere [46]. Raw gene expression data were background subtracted using Genome Studio and further processed using the Bioconductor R2.10.0 lumi-package [47]. The data were transformed by variance stabilization and quantile normalized. Probes significantly expressed (Illumina detection p-value < 0.01) in at least 3 samples from VSMC/C-VSMC and MSC/osteoblasts were considered as expressed and used for subsequent analysis, namely multivariate Principal Component Analysis (PCA).

Probes differentially expressed relative to the starting culture condition, i.e. day 0 of culture, were identified using the Bioconductor package ‘limma’ [48] with adjusted p-values (q-value) to reduce the false discovery rate. Differential expression was considered whenever a probe had a log_2_ fold-change >0.5 (up-regulation) or < -0.5 (down-regulation) relative to day 0 and a q-value <0.001.

### Data analysis: clustering, correlation and functional annotations

Differentially expressed probes were analyzed by k-means clustering using Gene Pattern (http://www.broadinstitute.org/cancer/software/genepattern/) [49]. Independent clustering analyses were performed for C-VSMCs and osteoblasts. The predictive power of k-means clustering was computed using FOM analysis in MultiExperiment Viewer v4.7 (http://www.tm4.org/) [50]. The maximum number of clusters and iterations was set to 15 and 50 respectively. From the FOM results we opted for 6 clusters (k = 6; Additional file 3: Figure S1) in both cell types. Differentially expressed probes within each of the groups identified by k-means clustering for C-VSMCs and osteoblasts were analyzed using DAVID Bioinformatics Resources 6.7 (http://david.abcc.ncifcrf.gov/) [51] to obtain a comprehensive description of the over-represented biological processes, cellular compartments and molecular functions. Redundant GO-terms were removed using REViGO (http://revigo.irb.hr/) [52].

In an independent targeted analysis, we matched expressed probes to GO-terms related to bone biology (GO:0031012, ECM; GO:0031214, biomineral tissue development; GO:0030282, bone mineralization; GO:0001503, ossification; GO:0045667, regulation of osteoblast differentiation; GO:0051924, regulation of calcium ion transport; GO:0016462, pyrophosphatase activity; GO:0030510, regulation of BMP signaling pathway). The Illumina probe/gene symbol information underlying each GO-term was retrieved using the Martview query from the BioMart open source tool version 0.7 (http://www.biomart.org). Genes underlying these GO-terms were subsequently used for correlation analysis essentially as described elsewhere [28, 53]. Briefly, we calculated the geometric mean of the intensities for each expressed probe set. The level of expression of each probe set was then determined relative to this geometric mean. The expression values were logarithmically transformed (on a base 2-scale) to impute equal weight to gene-expression levels with similar relative distances to the geometric mean. Deviation from the geometric mean was considered as differential gene expression. Similarities and dissimilarities between VSMC/C-VSMC and MSC/osteoblasts samples were visualized by Pearson’s correlation using Omniviz (BioWisdom Inc., version 6.0.1). As a control, similar correlation analysis were performed using randomly selected sets of expressed probes containing similar number of genes as the GO-terms analyzed.

### Gene expression validation at mRNA and protein level

RNA isolation was done as described above. cDNA synthesis and quantitative polymerase chain reaction (qPCR) were carried out as described elsewhere [54] except that the total amount of RNA was quantified spectrophotometrically using NanoDrop technology. Primer and probe sequences (5’ to 3’) were as follows: POSTN forward primer TGT GGA CAG AAA ACG ACT GTG TTA and reverse primer CGA TGC CCA GAG TGC CATA; TGFBI forward primer CTA CAT TTG GAG CCT GGA CA and reverse primer CCG GGT TAT GCT GGT TGTA; LEP forward primer ACA CAC GCA GTC AGT CTC CTC CAA and reverse primer AGG TCA GGA TGG GGT GGA GCC; SOST forward primer GAA TGA TGC CAC GGA AAT CAT and reverse primer CGG ACA CGT CTT TGG TCT CA; TAGLN forward primer GGC TGA AGA ACG GCG TGAT and reverse primer GAC CTT CAC CGG CTT GGA; ACTA2 forward primer GAG CGA GGC TAT TCC TTT GTGA and reverse primer ACG TAG CAC AGC TTC TCC TTG AT; PTGS2 forward primer GAA TCA TTC ACC AGG CAA ATTG and reverse primer TCT GTA CTG CGG GTG GAA CA; MGP forward primer CCT GCT CCT TCT CTC CAT TCTG and reverse primer TAG GAT TCC ATA CTT TCA TGA CAT TCG; ADAMTS1 forward primer GGA CAG GTG CAA GCT CAT CTG, reverse primer TCT ACA ACC TTG GGC TGC AAA and FAM/TAMRA probe CAA GCC AAA GGC ATT GGC TAC TTC TTCG; WNT5A forward primer GCT CCG CTC GGA TTC CTC and reverse primer CCA ATG GAC TTC TTC ATG GCG; GREM1 forward primer CGC CGC ACT GAC AGT ATG AG and reverse primer TCT TTT TCC CTT CAG CAG CC. All primers were purchased from Sigma-Aldrich.

For leptin measurements, medium was collected during VSMC/C-VSMC and MSC/osteoblasts cultures. Conditioned medium was collected after 78 h incubation with the cells. After centrifugation (5 min, 500 g), the medium was stored at −20°C until further analysis. Cell lysates were also collected to analyze the protein content of the corresponding cultures. Leptin was measured in 50 μl medium using the Human Leptin DuoSet DY398 ELISA kit (R&D Systems).

### Availability of supporting data

The gene expression data here analyzed is publicly available and can be retrieved from the Gene Expression Omnibus (GEO) at the National Center for Biotechnology Information (NCBI) under the accession number GSE37558 (data available at http://www.ncbi.nlm.nih.gov/geo/query/acc.cgi?acc=GSE37558).

## Electronic supplementary material

Additional file 1: Figure S3: Validation of the gene expression data. A selection of genes regulated in VSMC/C-VSMC and MSC/osteoblast was validated using qPCR in 2 independent donors (Donor 1 = main donor; Donor 2 = biological replication donor). Leptin expression was also confirmed at the protein level (ELISA) in the conditioned medium from VSMC/C-VSMC and MSC/osteoblast cultures (bottom right panel). (PDF 1 MB)

Additional file 2: Table S1: Number of probes differentially expressed in C-VSMCs and osteoblasts. Differential expression was calculated independently for both cell types and relative to their initial time point, day 0. The 4782 probes regulated were divided in three categories, identical or opposite regulation in the two cell types and cell-specific regulation. Only significantly regulated probes (q < 0.001) were considered. (XLSX 21 KB)

Additional file 3: Figure S1: Figure of merit (FOM) analysis to estimate k-means clustering predictive power in genes differentially expressed by C-VSMCs and osteoblasts. The lower the adjusted FOM value (y-axis) the higher the predictive power of the k-means algorithm. k =6 (dashed lines) was used for both cell types. (PDF 1 MB)

Additional file 4: Table S2: List of extracellular region probe/genes present in cluster 1 and 2 of C-VSMCs/osteoblasts, C-VSMCs only and osteoblasts only. (XLSX 59 KB)

Additional file 5: Table S3: List of ECM probe/genes differentially expressed and identically regulated in C-VSMCs and osteoblasts. (XLSX 64 KB)

Additional file 6: Table S7: List of biomineral tissue development and BMP signaling probes/genes used for correlation analysis in C-VSMCs and osteoblasts. (XLSX 54 KB)

Additional file 7: Figure S2: Expression profile of a selection of correlated biomineral tissue development genes and of anti-correlated BMP signaling genes during C-VSMC development and osteoblast differentiation. Expression is plotted as log_2_ fold-change relative to d0. Each line plotted represents a probe set. Probe/gene identifiers are provided in Additional file 6: Table S4. (PDF 1 MB)

## Abbreviations

ALP: Alkaline phosphatase
BMP: Bone morphogenetic protein
C-VSMC: Calcifying vascular smooth muscle cell
ECM: Extracellular matrix
FOM: Figure of Merit
GO: Gene Ontology
MSC: Mesenchymal stem cell
PCA: Principal component analysis
VSMC: Vascular smooth muscle cell.
