# Supplementary figures and images for: Calcifying vascular smooth muscle cells and osteoblasts: independent cell types exhibiting extracellular matrix and biomineralization-related mimicries
Source: BMC Genomics. 2014 Nov 7;15(1):965. doi: 10.1186/1471-2164-15-965 (PMC4247655; doi:10.1186/1471-2164-15-965)

Supplemental Figure 3.

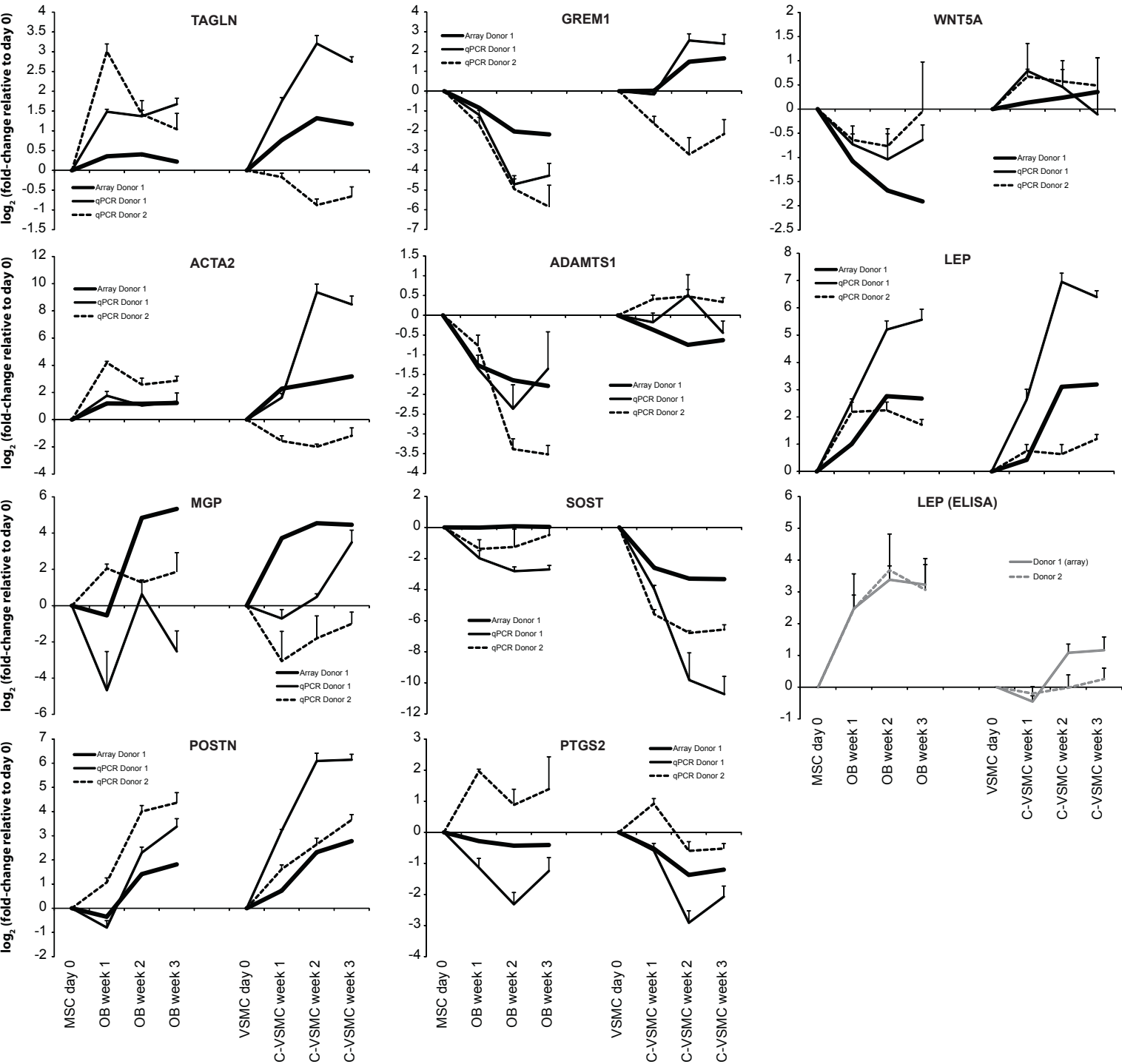

Supplement: Supplementary file 1 — Additional file 1: Figure S3: Validation of the gene expression data. A selection of genes regulated in VSMC/C-VSMC and MSC/osteoblast was validated using qPCR in 2 independent donors (Donor 1 = main donor; Donor 2 = biological replication donor). Leptin expression was also confirmed at the protein level (ELISA) in the conditioned medium from VSMC/C-VSMC and MSC/osteoblast cultures (bottom right panel). (PDF 1 MB) [file 12864_2014_6696_MOESM1_ESM.pdf]

# Supplementary Figure 1.

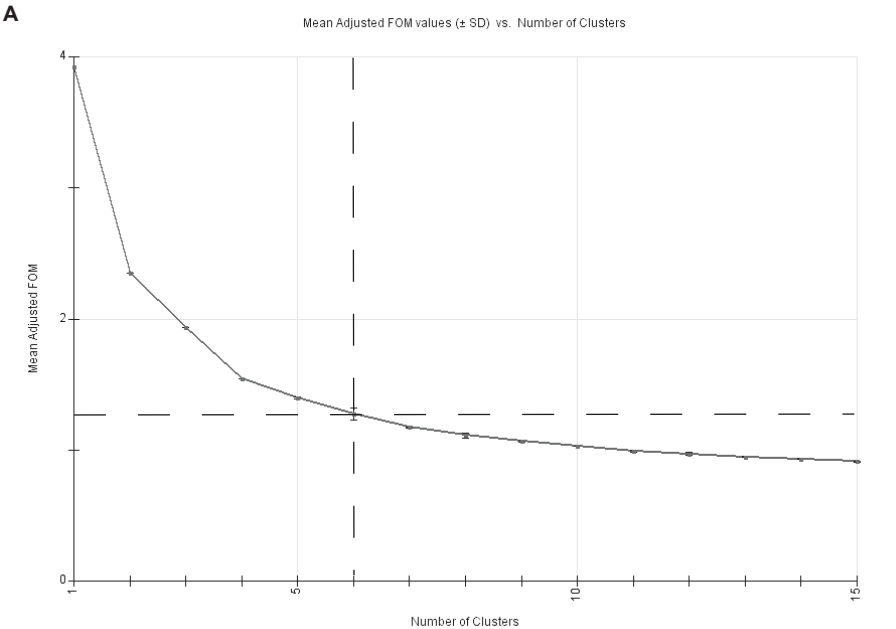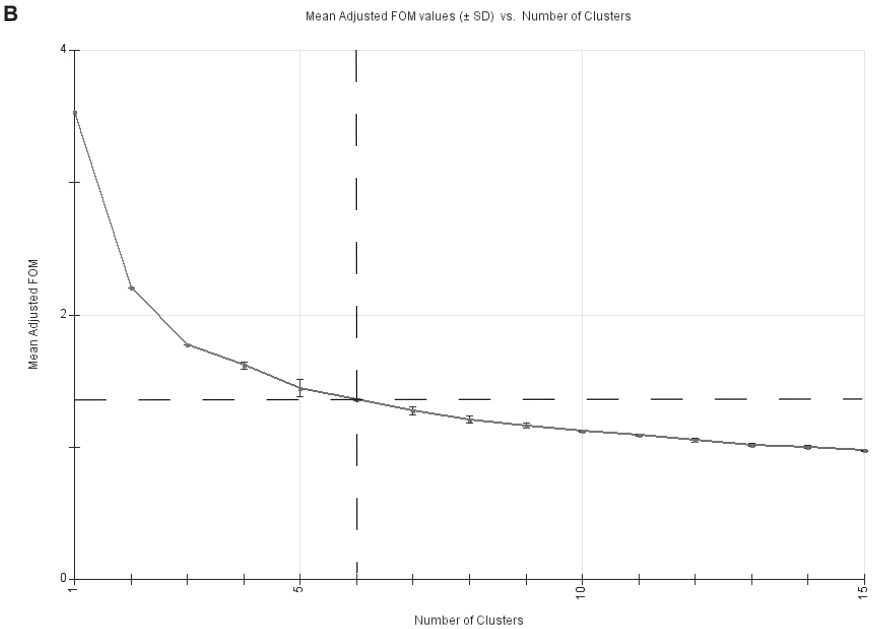

Supplement: Supplementary file 3 — Additional file 3: Figure S1: Figure of merit (FOM) analysis to estimate k-means clustering predictive power in genes differentially expressed by C-VSMCs and osteoblasts. The lower the adjusted FOM value (y-axis) the higher the predictive power of the k-means algorithm. k =6 (dashed lines) was used for both cell types. (PDF 1 MB) [file 12864_2014_6696_MOESM3_ESM.pdf]

# Supplementary Figure 2.

**A**

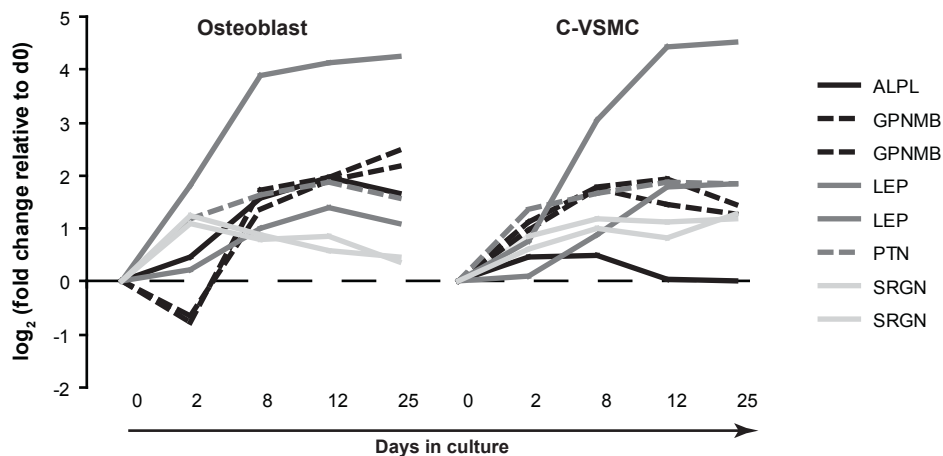

**B**

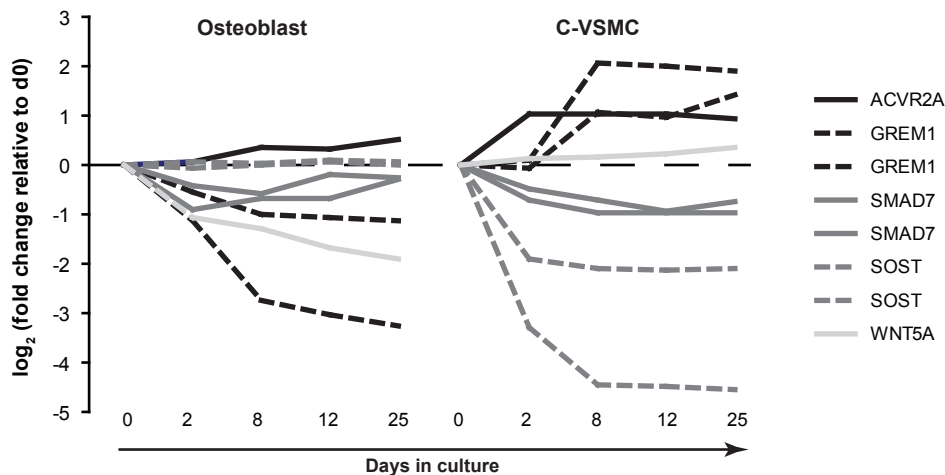

Supplement: Supplementary file 7 — Additional file 7: Figure S2: Expression profile of a selection of correlated biomineral tissue development genes and of anti-correlated BMP signaling genes during C-VSMC development and osteoblast differentiation. Expression is plotted as log2 fold-change relative to d0. Each line plotted represents a probe set. Probe/gene identifiers are provided in Additional file 6: Table S4. (PDF 1 MB) [file 12864_2014_6696_MOESM7_ESM.pdf]
